# Effect of Cellulose Ether and Starch Ether on Hydration of Cement Processes and Fresh-State Properties of Cement Mortars

**DOI:** 10.3390/ma15248764

**Published:** 2022-12-08

**Authors:** Edyta Spychał, Piotr Stępień

**Affiliations:** Faculty of Civil Engineering and Architecture, Kielce University of Technology, 25-314 Kielce, Poland

**Keywords:** cellulose ether, starch ether, VMA, cement mortar, consistency, water retention, application properties, setting time, hydration process, calorimetric measurements

## Abstract

The production of factory-made mortars is a multicomponent system. Viscosity-modifying admixtures (VMAs) are an inherent ingredient of these materials. The correct choice of the amount and type of these admixtures is important from the practical and scientific points of view. In this article, the use of cellulose ether (CE) and starch ether (SE) in cement pastes and mortars is studied. This research focuses on the hydration process and fresh-state properties of mortars because this subject determines the correct choice of the amount and type of admixture used, and the results determine the application and properties of hardened mortars. Polymers were added in the range from 0.056% to 0.22% in relation to the dry ingredients of the mortar. The research showed that cellulose ether had the greatest impact on the consistency, air content, bulk density, and water retention of ordinary dry-mix mortars. On the other hand, starch ether affected the hydration process, delaying the setting and hardening processes much more than cellulose ether. The action of these admixtures rose with the increase in the amount of polymer used in different ways (depending on the type of ether).

## 1. Introduction

Chemical admixtures are widely used to modify mortars and concretes in order to obtain standard or special properties. Mortar formulations quite often comprise from a few to over a dozen different ingredients, so the development of such recipes for mortar requires great knowledge and practice. Even small amounts of some admixtures can have a significant effect on the properties of fresh and hardened mortars. Among the wide range of these substances used in the production of factory-made masonry, plastering, and adhesive mortars, there are polymer admixtures, which include, among others, cellulose ethers and starch ethers [1,2,3,4,5,6]. The way in which both of these admixtures affect the properties of mortars is varied and depends on many factors [7,8,9,10,11,12]. The correct selection of the type of admixture and its amount consists of a compromise between obtaining the appropriate consistency and rheological properties (ensuring adequate fluidity, plasticity, and nonsegregation), good workability, and high water retention, which ensures the appropriate conditions for hydration, as well as the mortar setting and hardening processes, and reduces shrinkage deformation.

Cellulose is a natural polysaccharide. It commonly occurs as the basic construction material in plant tissues. Pure cellulose is a white, water-insoluble substance, which must be further processed to be used as a chemical admixture. Cellulose ethers arise from a chemical industrial process. The plants etherified to obtain polymer admixtures include pure cotton (about 90% cellulose), wood (about 40–55% cellulose), and straw or reed (30–40% cellulose) [13,14,15,16,17]. The production of these admixtures consists, inter alia, of grinding, activation with NaOH aqueous solution, a heterogeneous reaction with an etherifying agent, neutralization, the isolation of crude cellulose ether, purification by extraction of the salts and by-products, drying, and confectioning [16,17]. These cellulose derivatives control the water retention in fresh-state mortars and can increase adhesion power [7,12,18,19,20,21,22,23,24,25]. They influence the rheological properties (viscosity and yield stress). Their purpose is the improvement of the workability of mortars [3,7,18,26]. Additionally, cellulose ethers affect the delay of hydration processes, as well as the setting and hardening processes [3,10,27,28,29], and decrease compressive strength [3,5,30,31,32,33].

Starch is a natural polymer derived from the polysaccharide group. It is most often obtained from potatoes or corn. It is found in tubers, seeds, and plant roots. The main component of starch is amylopectin [9,11,34,35]. Due to its physicochemical properties, the possibilities of using starch are quite limited. Therefore, it undergoes physical and chemical modifications that give it desired parameters, such as texture, solubility, and consistency. Starch ethers are added to dry-mix mortars or concretes. They can modify the rheology of fresh-state mortars by bridging flocculation. In general, starch ethers can enhance workability and reduce segregation of the components of mortar [11,34]. As introduced in previous publications [5,8], starch ether has shown a considerable delaying effect without adversely impacting compressive strength (unlike cellulose ether admixtures).

In the literature, one can usually find research on the properties of mortars modified with only one admixture—either starch ether or cellulose ether. Analyses often concern the influence of one type of admixture on the properties of pastes or mortars (for example, taking into account various chemical modifications, quantity, and viscosity) [4,6,10]. Some studies focus on the detailed analysis of a selected problem. In articles [10,20,27,28], the impacts of cellulose ethers on C_3_A or cement hydration have been examined. These works have shown that cellulose ether has an influence on these processes. This polymer leads to a gradual slowing down of hydration dependent on cellulose ether chemistry. Water retention and rheological measurements have been tested in various reports [12,22,26]. The influence of cellulose ether on water retention is more noticeable than when using starch ether. Research has also tested mortars consisting of combinations of several different ingredients, one of which being either cellulose ether or starch ether [2,4,18,30].

Some research on pastes and mortars can be found in which the authors compare the actions of different VMAs. In [5], mortars with four types of chemical admixtures, including, among others, cellulose ether and starch ether, were tested. The effects of these polymers on the working and mechanical properties of dry-mixed mortars and the relationships between the performances were studied. The amount of admixtures used was in the range of 0.02–0.08% (by mass in relation to all the dry ingredients of the mortar). Consistency, water retention, and 28-day compressive strength tests were carried out for mortars with different amounts of each of the admixtures. On the other hand, the setting time was determined for samples with selected amounts of ethers (three amounts of cellulose ether and one dose of starch ether), which did not allow the full assessment of the setting and hardening processes. As can be seen, the effect of cellulose ether was more varied in the case of the consistency test. The consistency for mortars modified with cellulose ether ranged from 88 mm to 98 mm, whereas for mortars modified with starch ether, it ranged from 60 mm to 110 mm. The water retention results were similar for both ethers (from about 85% to 100%). As the admixtures were added, the setting time increased. In [36], the influences of two polysaccharides were compared. The research reported different properties between welan gum and cellulose ether; fluidity after 0, 60, and 120 min; 3-day and 28-day compressive strength; 7-day and 28-day adhesive strength; rheological properties; and zeta potentials. Mortars modified with welan gum thickened instantly and increased after 60 min, but the fluidity was still smaller than that in the reference sample. After a longer time, the fluidity dropped. The fluidity of mortar modified with cellulose ether after 0 min was the same as that of the reference sample (without admixture), but the flow decreased with time. Comparing the fluidity loss measurements between 0 min and 180 min, the mortar without admixture had 25.3% fluidity loss, but the results of modified mortars were only about 10.2–11.5%. The results for compressive strength and adhesive strength were different for cellulose ether and welan gum. For example, the strength loss of mortar with cellulose ether amounted to about 47%, whereas mortar with welan gum reached about 7%. On the other hand, the adhesive strength of mortar with welan gum was similar to that of the reference mortar, but the tested parameter was doubled at 7 days and 14 days for mortar with cellulose ether. In [26], the authors reported the influences of cellulose ether, starch ether, and guar gum on the rheological behavior of render mortar and their relationships with water retention. The amounts of admixtures used were from 0.03% to 0.24% (cellulose ether) and from 0.03% to 0.12% (starch ether and guar gum). Increase in the amount of cellulose ether or guar gum resulted in a significant increase in the water retention value (in the range from about 80% to about 100%), which was not as noticeable when adding starch ether (in the range from about 74% to about 86%). Starch ether increased the gel feature of mortars and had more impact on cohesion, while the other two admixtures decreased the gel-like responses of the tested samples. The research concentrated only on the parameters of mortars in the plastic state (application properties), and the setting and hardening processes were not taken into consideration.

The goal of the present work is the study and comparison of the uses of two types of viscosity-modifying polymer admixtures, which have important roles in dry-mixed mortars. The variables in the study are the type of admixture used and its amount. The paper reports different properties between mortars with cellulose ether and starch ether, as well as consistency, air content, and bulk density. The WRV value is defined as water retention measurements tested after, 10, 20, and 30 min. On this basis, the application properties of these materials are analyzed. Additionally, the influences of admixtures on the setting and hardening processes of the pastes are examined on the basis of calorimetric measurements and setting-time research. Setting-time research gives information about the initial, final, and total setting times, but the calorimetric method allows the full introduction of the hydration process in the first several hours of research. These parameters can allow a full understanding of the actions of cellulose ether and starch ether, which is necessary for the correct choice of the type and amount of admixture used. Additionally, if starch ether is added to dry-mix mortars with cellulose ether, understanding of its action and choosing the amount of starch ether should be preceded by research on the separate effect of each ether on the properties of pastes and mortars. In the present work, we assess how the same amount of cellulose ether and starch ether admixture affects the tested properties, as well as whether the scope of their activity is the same. In addition, the statistical influence of the amount of admixture on the tested parameters of pastes and mortars is verified, and on this basis, trends in changes of the tested parameters are determined. In summary, the article supplements the knowledge on the scope of operation of cellulose ether and starch ether based on the standard and nonstandard properties of pastes and mortars in the plastic state, as well as during the setting and hardening processes. On the basis of the research performed, it can be stated how the properties of pastes and mortars change, taking into account variable doses of admixtures, and it can be assessed how the same amount of both cellulose ether and starch ether affects the tested parameters, which complements the knowledge on the scope of operation of these polymers.

## 2. Materials and Methods

### 2.1. Materials

#### 2.1.1. Cement, Fine Aggregate, and Water

**Figure 1:**
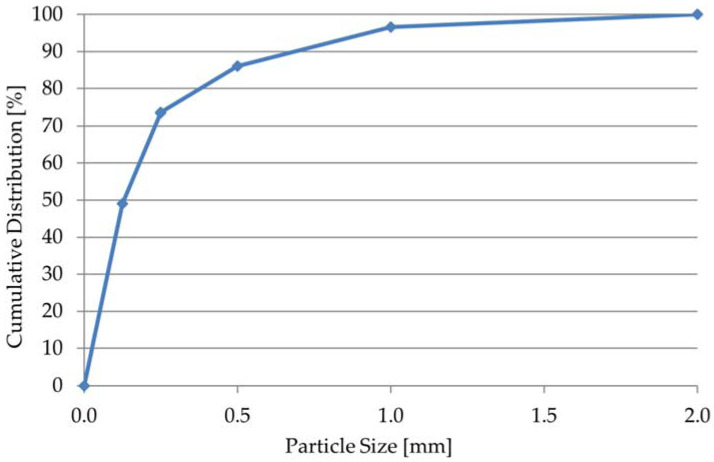
Particle size distribution of chalcedonite sand.

CEM I Portland cement of the 42.5 R class (Cemex, Chełm, Poland), natural chalcedonite sand with a fraction of 0.1–1.0 mm as a fine aggregate (Crusil, Inowłódz, Poland), and tap water were the basic ingredients in the mortars. Selected physicochemical properties of the cement are given in Table 1. The grain size distribution of the chalcedonite sand is shown in Figure 1. Based on the grain size distribution curve, it was established that the chalcedonite sand particles contained 49% ranging up to 0.125 mm and 47% in the range of 0.125–1.0 mm.

#### 2.1.2. Polymer Admixtures

The compositions of mortars were modified with the following commercial polymers: cellulose ether or starch ether as VMAs. Admixtures recommended by the producer for the production of cement-based mortars were selected for this research. The physical and chemical properties of CE and SE are shown in Table 2 and Table 3.

### 2.2. Sample Preparation

The amounts of binder, water, and sand were constant for all the mortars. The proportions by weight were one part cement to three parts fine aggregate. The amount of cellulose ether and starch ether used varied, and it constituted from 1 g to 4 g (from 0.056% to 0.22% in relation to the dry ingredients of the mortar—cement and sand). The water-to-cement ratio was 1.33 for all the mortars. The amount of water in the reference sample (REF) was chosen to obtain a constant flow table of 165 ± 5 mm. The mix proportions of the cement mortars are shown in Table 4. The control sample (REF) without admixture was prepared. The next eight specimens were modified with cellulose ether (CE1–CE4 symbols) or starch ether (SE1–SE4 symbols).

The binder, fine aggregate, and water were weighed with an accuracy of 0.1 g, and the polymer admixtures were weighed with an accuracy of 0.0001 g. Water was added to the weighed, pre-mixed components, and everything was mixed mechanically for 3 min with an interval of 30 s in the middle of mixing. The samples prepared in this way were used for testing consistency, bulk density, air content, and WRV values.

Paste samples were produced by adding the appropriate amount of polymer admixture (from 0 to 4 g) to 500 g cement and mixing (with water) mechanically in the same way as mixing mortars. Then, the setting-time test was performed for each of the nine samples. For CE and SE, eight pastes were prepared with the following proportions: 5 g cement, 2.5 mL distilled water, and 0, 0.01, 0.02, 0.03, or 0.04 g of admixture. Then, the samples were prepared for calorimetric measurements. The ninth reference sample (REF) did not contain any admixture. The hydration process was finished after 72 h. The setting time and the heat of cement hydration were measured for the pastes with a water-to-cement ratio of 0.5. A constant ratio of 0.5 for water-to-binder for these two types of research was chosen due to the possibility of comparison of the obtained results with the literature data.

Preparation of samples and testing were performed in a laboratory at a temperature of 20 ± 2 °C and a relative air humidity of 65 ± 5%.

### 2.3. Methods

After mixing the components, the consistency of the fresh mortars was tested by two methods. The flow table was determined by the PN-EN 1015-3 method [37] as the spread diameter of the mortar sample. The cone slump was tested according to the PN-B-04500 standard [38] as measuring the depth of the cone in mortar.

The air content was standardized by a pressure method according to PN-EN 1015-7 [39]. The final results were given with an accuracy of 0.5%.

The bulk density of fresh mortars was determined according to PN-EN 1015-6 [40]. The final results were given with an accuracy of 10 kg/m^3^ for each sample.

Water retention research was conducted using a nonstandard method following [41]. The water retention value was the result obtained from the difference between the value of 100% and the value of the water loss. The water loss was calculated on the basis of water absorption by filter papers in short-term contact with the fresh mortars, and measurements were performed after 10, 20, and 30 min of contact of the paper with the samples of mortar. Immediately after mixing the components, two mortar samples were molded each time. The water retention value was labeled WRV, with WRV10 being highlighted (water retention measured after 10 min), as well as WRV20 and WRV30 (water retention measured after 20 and 30 min, respectively).

The setting times, as well as the beginnings and ends of the setting times, of cement pastes were measured according to the PN-EN 196-3 standard [42].

The rate of cement hydration and the total heat evolution were assessed by heat evolution with a nonadiabatic–nonisothermal microcalorimeter. The measurements were obtained using a BT 2.15 low-temperature differential scanning microcalorimeter (Setaram, Plan-Ies-Ouates, Geneva, Switzerland). The samples were prepared by blending the dry components with distilled water. The pastes were stored in small, sealed PE zip-bags and placed in the calorimeter. The changes in heat evolution were recorded every 30 s for a period of 72 h of measurement and saved automatically in a computer’s memory. On the basis of these data, the heat evolution curves and the total heat evolution curves as a function of time for all pastes were obtained. Additionally, the degree of hydration was calculated and compared using [43] and the following pattern:

(1) $$SH=\left(\frac{x_{1}}{x_{2}}\right)\cdot 100,$$

where *SH* is the degree of hydration, *x*_1_ is the value measured after a specified hydration time, and *x*_2_ is the value measured after full hydration or after a specified hydration time taken as a point of reference.

In this article, the cumulative amount of heat hydration after 72 h was assumed as the result of the *x*_2_ value read for REF. However, value *x*_1_ was applied as the cumulative amount of heat hydration after 12, 24, 36, 48, 60, and 72 h, so Formula (1) took the following form:

(2) $$SH=\left(\frac{Q_{t}}{Q_{72h}}\right)\cdot 100,$$

where *SH* is the degree of hydration; *Q_t_* is the cumulative amount of heat hydration after 12, 24, 36, 48, 60, 72 h for each sample; and *Q*_72h_ is the cumulative amount of heat hydration after 72 h for the reference sample (REF).

## 3. Results and Discussion

### 3.1. Consistency Measurements

**Figure 2:**
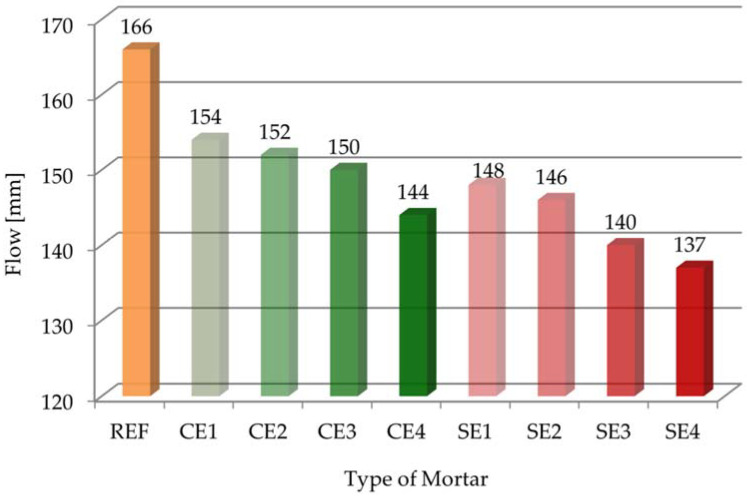
Flow of tested mortars.

The results of flow for all the samples are presented in Figure 2 (consistency measurements according to the standard of [37]).

The flow of the reference mortar (REF) was 166 mm. The results of the studies on the flow of mortars modified with cellulose ether ranged from 154 mm down to 144 mm (decreasing from 7% to 13%). The flow value of the CE4 sample was 144 mm, which was about 13% smaller than that of the REF sample. The results of the studies on the flow of samples modified with starch ether ranged from 148 mm down to 137 mm (decreasing from 11% to 17%). The flow value of the SE4 sample was 137 mm, which was about 17% smaller than that of the REF sample. As can be seen, the addition of even a small amount of starch ether significantly decreased flow. The consistency decreased as the amount of admixture increased, regardless of the type of admixture, but in the case of the action of starch ether, this effect was more noticeable. Both cellulose ether and starch ether have the effect of reducing flow, which must be taken into account when selecting the appropriate amount of admixture for a mortar. By analyzing all the results, it can be said that the used admixtures in amounts of 0.056–0.22% behaved as thickeners. These test results are consistent with those obtained in various publications [2,6,44]. Additionally, by comparing all the results, it can be seen that all the mortars, except for SE4, were characterized by plastic consistency (according to the standard of [40]). The SE3 sample had a dense plastic consistency. Flow is a very important parameter to characterize the workability and plasticity of mortar, governing mix behavior during application on walls and placement in construction [36].

**Figure 3:**
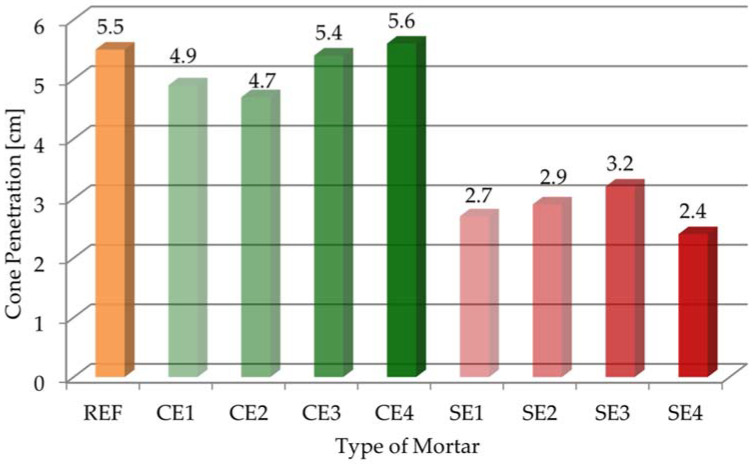
Cone penetration of tested mortars.

The results of cone penetration for all the mortars are presented in Figure 3 (consistency measurements according to the standard of [38]).

The cone penetration of the reference mortar (REF) was 5.5 cm. The results of the studies on the consistency of mortars modified with cellulose ether ranged from 4.7 cm to 5.6 cm, and the consistency of mortars modified with starch ether ranged from 2.4 cm to 3.2 cm. It is clearly visible that starch ether reduced the cone penetration (by even twice as much compared to the reference sample). On the other hand, the effect of cellulose ether did not change the consistency significantly.

The obtained test results are also worth supplementing with visual observations of the samples during the consistency test using the flow table method. The mortar with 4 g of cellulose ether (CE4) stuck to the walls of the mold. It can be assumed that its dosage was so high that it could cause problems with mortar application. This phenomenon was not observed for the mortar with the same amount of starch ether (SE4). On the other hand, the SE4 sample was more “dry” and less consistent in whole mass than the CE4 sample. It can be assumed that the doses of 0.22% of each ether were too much. In this case, the application of these admixtures seemed ineffective.

### 3.2. Air Content of Mortars

**Figure 4:**
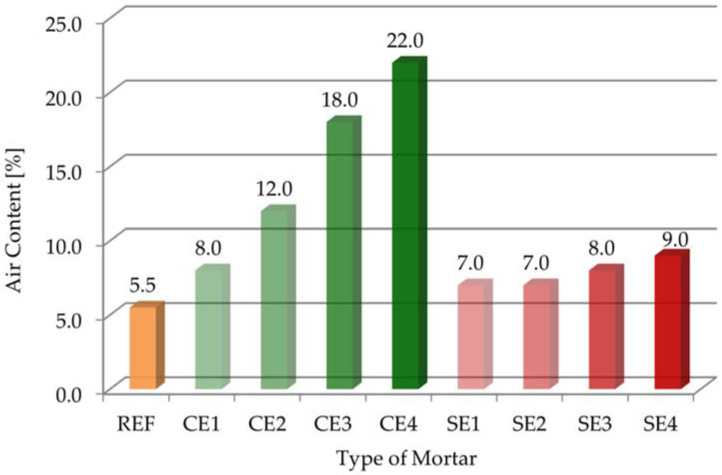
The results of air content for tested mortars.

Figure 4 presents the results of air content for all the mortars.

The results presented on Figure 4 show that the REF control mortar had the smallest air content, which was 5.5%. The modification of mortars with cellulose ether significantly increased air entrainment. Mortars that presented the highest air content values (C2–C4) were prepared with amounts of cellulose ether from 2 to 4 g. In the case of the CE4 sample, there was a four-fold increase in the air content. These trends are similar with those obtained in previous reports [23,24]. Compared with the reference sample (REF), incorporating starch ether brought a bit higher aeration. Admixtures of starch ether did not have as big an influence on air content as those of cellulose ether. The percentages of entrained air for mortars modified with starch ether are comparable to results obtained in previous articles [6,25,45].

### 3.3. Bulk Density Measurements

**Figure 5:**
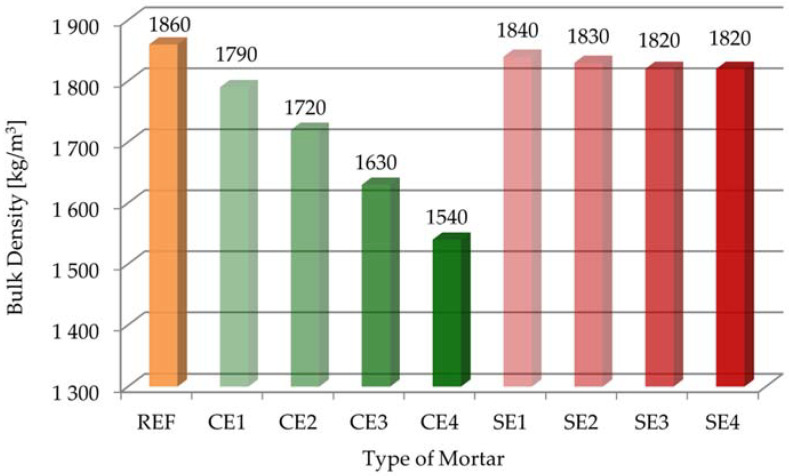
Bulk density of tested mortars in the plastic state.

Figure 5 presents the results for bulk density for the reference mortar and mortars modified with polymer admixtures in the plastic state.

As can be seen, the bulk density values of all the mortars with cellulose ether and starch ether were smaller than that of the reference sample. This parameter of the REF mortar was 1860 kg/m^3^. The parameter ranged from 1540 kg/m^3^ (CE4 sample) to 1790 kg/m^3^ (CE1 sample) for mortars containing cellulose ether. Adding 4 g of cellulose ether even caused a 17% reduction in bulk density. The parameter ranged from 1820 kg/m^3^ (SE4 sample) to 1840 kg/m^3^ (SE1 sample) for mortars containing starch ether. The addition of the admixture of starch ether led to a slight change in the bulk density. On the other hand, higher the content of cellulose ether in the mortar composition caused definitely smaller bulk density, which favorably affected the output. This is especially important from economic and practical points of view. The entrained air had an effect on the reduction of density of the mixture, which is important in terms of improved workability and, especially, during the application of mortar on walls or ceilings [7,46]. Figure 4 and Figure 5 show that the bulk density decreased with increasing air content in the tested samples because the variation in bulk density was essentially due to the change in the air content of mortars.

### 3.4. Water Retention of Tested Mortars

**Figure 6:**
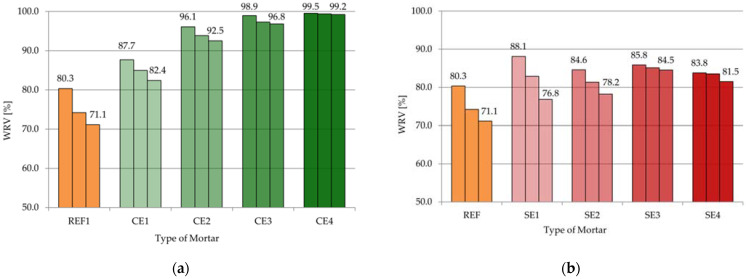
Water retention values (**a**) for mortars modified with cellulose ether and (**b**) for mortars modified with starch ether.

Table 5 and Figure 6a,b present the results of the water retention values for WRV10, WRV20 and WRV30 (tests after 10, 20, and 30 min, respectively) for the tested mortars. Each time, the graph shows three results for each of the mortars, showing the water retention after 10, 20, and 30 min. The water retention mechanism is one of the key properties of mortar, so this research is important for rating mortars, as well as in the choice of the type and amount of VMA used. High water retention is especially crucial when a mortar is applied in thin layers on highly absorbent substrates when changing thermal and humidity conditions during bricklaying or plastering occur. In these situations, appreciable water loss occurs through evaporation and suction [3,14].

The results of water retention for REF were 80.3% after 10 min measurements, followed by 74.2% and 71.1% after 20 and 30 min, respectively. Thus, the mortar without admixtures obtained the lowest parameters. This sample had the smallest water retention. The water loss after 30 min was about 29%. As shown in Table 5 and Figure 6a, increased amounts of cellulose ether admixture increased the WRV from 87.7% to 99.5% (WRV10), from 84.9% to 99.4% (WRV20), and from 82.4% to 99.2% (WRV30). Finally, the loss of water for the mortar with the smallest amount of CE after 30 min was equal to 17.6%, but for the mortar with the biggest amount of CE, it was 0.8%. The WRV values are similar to the results of a previous report [26]. In [47], the authors claimed that the working mechanism of cellulose ether relied on two separate effects: firstly, its water sorption capacity and, secondly, the formation of hydrocolloidal-associated 3D polymer networks. On the other hand, the WRV10 value ranged from 83.8% (SE4) to 88.1% (SE1), the WRV20 value ranged from 81.3% (SE2) to 85.1% (SE3), and the WRV30 ranged from 76.8% (SE1) to 84.5% (SE3). The water retention values were less for mortars modified with starch ether compared with the parameters for mortars with cellulose ether. The amount of starch ether used only allowed an increase in water retention to a certain extent (at a lower level than in the case of the use of cellulose ether). This behavior was also observed by the authors of a previous publication [26]. The amount of SE can be critical in this case, as results and conclusions have shown in previous publications [6,26]. They have claimed that only the highest amounts of this admixture lead to higher water retention than mortars without modification (in previous research, for concentrations of SE above 0.3% *w*/*w* polymer: dry mortar).

In summary, the effect of water retention was more visible and noticeable for mortars modified with CE than for samples modified with SE (comparing the amounts of admixtures used in the tests).

**Figure 7:**
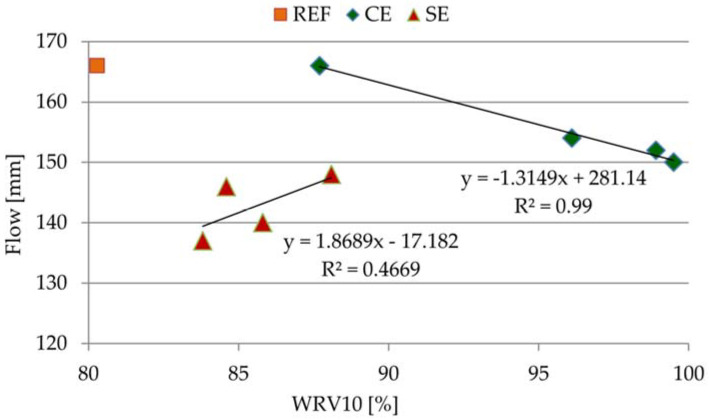
Correlation between flow of mortars and WRV value.

In Figure 7, the correlation between the consistencies of all the tested mortars (consistency) and water retention values after 10 min was introduced. As a consequence, the conclusion of Figure 7 is that water retention was indirectly related to the flow of mortars. While in the case of cellulose ether, it could be said that high water retention values applied to mortars with less consistency (especially when adding from 2 g to 4 g of admixture), in the case of starch ether, it is too general and approximate a conclusion.

### 3.5. Setting Times of Tested Pastes

**Figure 8:**
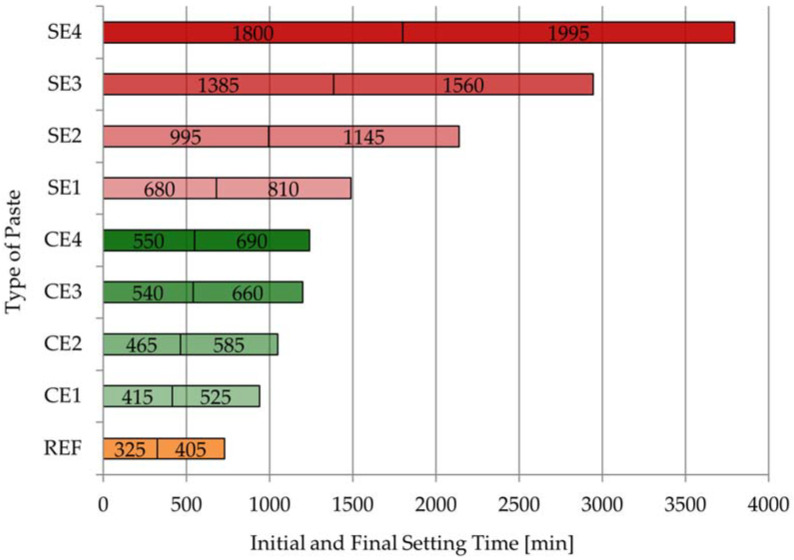
The initial and final setting times for all tested pastes.

**Figure 9:**
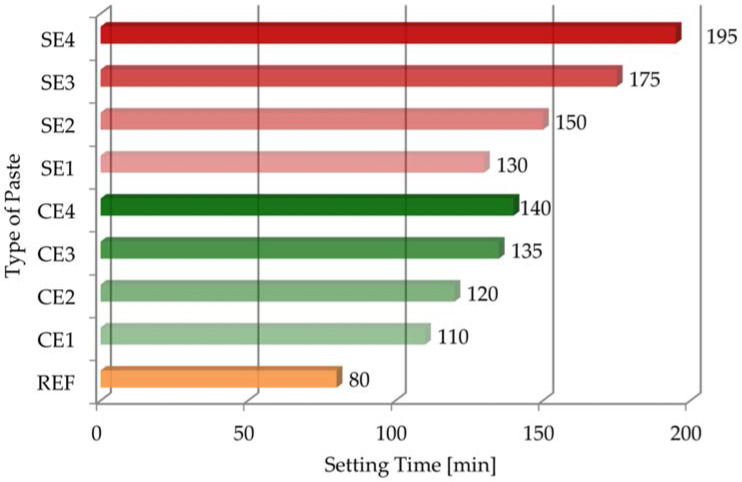
The results of setting time for all tested pastes.

Figure 8 and Figure 9 present the results of the initial and final setting times, as well as the setting times for all the pastes.

The initial setting time for REF was noted after 325 min, but the final setting time was after 405 min, so the setting time for the reference mortar was 80 min. Both cellulose ether and starch ether influenced the binding and hardening processes of the tested pastes. However, this effect was more noticeable with starch ether. Especially large differences in the impacts of the ethers can be observed by comparing the results in Figure 8. The beginning of the setting time for CE1 was 415 min, but the end of the setting time was 525 min; these parameters for the SE1 mortar were 680 min and 810 min, respectively. On the other hand, the beginning of the setting time for CE4 was 550 min, and the end of the setting time was 690 min, but these parameters for the SE1 mortar were 1800 min and 1995 min, respectively. There was an even bigger difference of three times in the parameters between pastes with different polymers. The setting time for REF was 30 min shorter than that for the CE1 sample and 60 min shorter than that for the CE4 sample. In the case of starch ether, these differences were greater. The setting time for REF was 50 min shorter than that for the SE1 sample and 115 min shorter than that for the SE4 sample. In conclusion, the starch ether admixture had a significant effect on the setting processes of the tested pastes, which is consistent with the findings of similar studies [8,48]. For example, the initial setting time for the reference mortar was 150 min, and the final setting time was 240 min, but mortar modified with 1% starch ether (calculated by mass of cement) had an initial setting time of about 225 min and a final setting time of 420 min. Moreover, the dosage of starch ether had a significant role in regulating the setting times of pastes, which was more noticeable for the samples with SE than with CE. Delay of setting processes has obtained by the authors of previous publications [19,31]. It was confirmed that the amount and viscosity of cellulose ether had influences on setting time. The extended setting time of the pastes was directly related to the properties of the admixtures used. One of these parameters was water retention. High WRV values of the modified mortars affected water retention because the water remained in the mortars for a longer time.

### 3.6. Heat of Hydration of Pastes

**Figure 10:**
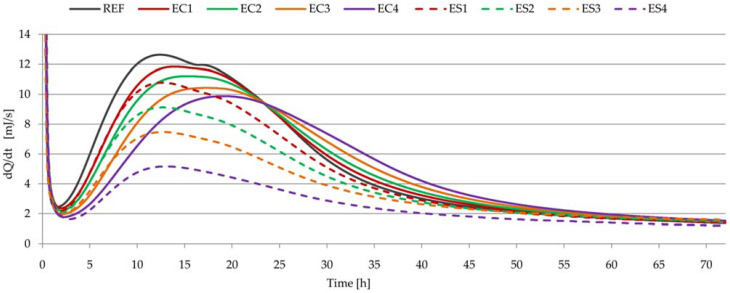
Heat evolution curves as a function of time for all pastes.

**Figure 11:**
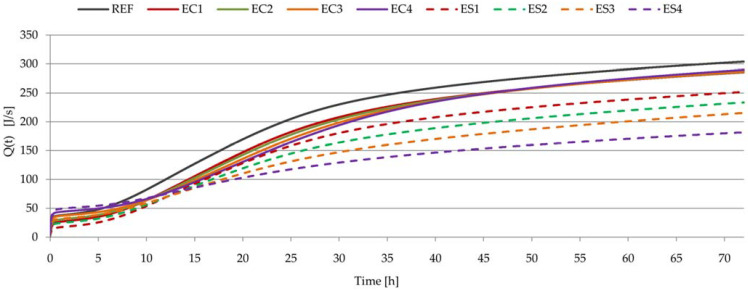
Total heat evolved as a function of time for all pastes.

Figure 10 shows the heat evolution curves of cements with different amounts and kinds of polymer admixtures, and Figure 11 shows the total heat evolution of these pastes. In Table 6, some parameters relating to heat evolution curve characteristics are noted, including the maximum value and position, as well as heat evolution after 12, 24, 36, 48, 60, and 72 h. Additionally, the degree of hydration (Table 7) was calculated based on Formulas (1) and (2), taking into account the results from Figure 11 and Table 6 (based on the amount of heat evolution).

On the basis of the calorimetric measurements shown in Figure 10 and Figure 11, it can be stated that the amount of ether and the type of ether delayed the hydration process. These admixtures did not change the typical profile of the curve of cement hydration, although two maxima can be noted for the reference sample (between 12 and 20 h), while only one maximum is clearly visible for all the modified samples. The polymers caused an extension of the delay, suppression of the main heat release peak, and delay of the induction time (Figure 10). The biggest heat evolution in the first 72 h of hydration and the biggest total heat evolution were noted for the REF sample. Cellulose ether and starch ether caused lower heat evolution, and the total heat evolution values were smaller. This effect was easily visible at higher admixture dosages. The hindering effect was more pronounced in cases of pastes with SE than in pastes with CE. These conclusions confirm observations and research previously reported in the literature [19,25,28]. Incorporating cellulose ether mainly delayd the early hydration processes of cement pastes but showed negligible influence on mid–late hydration [25]. These conclusions are also confirmed by the supplementary calculations: the degrees of hydration of pastes with cellulose ether were at a similar level as that of the reference paste. A different effect was noted in the case of the use of starch ether.

### 3.7. Complementary Analysis of the Obtained Research Results

A supplementary analysis of the obtained research results is introduced in this chapter, including an assessment of the effects of the amount and type of polymer VMA on the selected properties of mortars and pastes. Table 8, Table 9, Table 10 and Table 11 summarize the parameters that allow for the evaluation of the statistical influence of the amount of each admixture on the tested properties based on the classification presented in publication [49]. A comparison of the properties of polymer admixtures in the tested pastes and mortars is introduced in Table 12 and Table 13. Trends of changes in the obtained results were determined depending on the amount and type of admixture used.

## 4. Conclusions

In this paper, research was carried out on mortars modified with cellulose ether and starch ether. The purpose of this study was to evaluate the effect of the amount and type of polymer admixture used on selected properties of cement pastes and mortars. An evaluation was conducted on the fresh-state properties of mortars and the hydration processes of pastes. It was found that the amount, as well as the type of ether used, affected the properties of the tested materials differently. The following particular conclusions were drawn from this research:

1. An increase in the amount of polymer admixture resulted in a decrease in flow. Starch ether had a bigger effect on consistency changes. On the other hand, cone penetration was almost the same for mortars modified with cellulose ether and mortars without admixture. Starch ether had a significant influence on the reduction in cone penetration.
2. It was noted that adding cellulose ether admixture could affect the air content, even quadrupling it. In contrast, mortars modified with starch ether had almost two times bigger air content compared with the reference sample.
3. Increase in the dosages of both admixtures decreased bulk density, but the use of cellulose ether was of greater importance, so the workability of these mortars was the biggest. Correlation between the bulk density and air content of the tested mortars showed decreases in bulk density with increases in air content. On the other hand, when air content increased, compressive strength decreased.
4. Mortars modified with cellulose ether and starch ether were characterized by high water retention values. For example, the water retention after 10 min increased from 80.3% with a 0% dose of cellulose ether to 99.5% with 0.22% CE (CE4). Generally, the effectiveness of cellulose ether increased with increase in the dose of this admixture. The WRV value of mortars modified with starch ether ranged from 83.8% (SE1) to 88.1% (SE4). The water loss after 30 min was almost 29% for the reference mortar (REF), which was a negative phenomenon. Mortars modified with 3 and 4 g of cellulose ether had the smallest water loss values. The flow test results correlated with the water retention measurements, especially in the case of the cellulose ether admixture.
5. It was clearly noticeable that both admixtures delayed the initial and final setting times, as well as lengthened the total setting time. Bigger differences in the results between the reference and the modified samples were obtained as a result of adding starch ether. A larger amount of admixture extended and delayed the tested parameters.
6. Cellulose ether and starch ether both led to a gradual slowing of cement hydration and decreased heat evolution, which was also confirmed by the calculation of the degree of hydration.
7. In addition to the results of the cone penetration tests, the statistical influence of the amount of admixture used was noted for the tested parameters.
8. Polymer admixtures acted with different intensity levels on the tested properties of the pastes and mortars. Additionally, the tendencies of changes in the test results were different for each ether.

Due to the differential effects of cellulose ether and starch ether on the properties of mortars in the plastic state, as well as on the setting processes of pastes, the selection of the type of polymer and the amount of admixture used should be preceded by thorough analysis, which allows for obtaining the desired properties of a finished product (optimal properties for factory-made mortars).

## Figures and Tables

**Table 1:** Selected physicochemical properties of cement.

Property	Result
Initial setting (min)	173
Final setting (min)	237
Specific surface area (m^2^/kg)	374.6
Loss on ignition (%)	3.24
2-day compressive strength (MPa)	25.6
28-day compressive strength (MPa)	54.3
S0_3_ (%)	2.98
Cl (%)	0.079

**Table 2:** Physical and chemical properties of the used cellulose ether.

Polymer Type	Cellulose Ether (CE)
Appearance	Whitish powder
Chemical modification	Methyl hydroxyethyl cellulose (MHEC)
Eterification	Standard
Viscosity (according to Höppler)	15,000 mPa·s
Viscosity (according to Brookfield RV ^1^)	11,000–15,000 mPa·s
Humidity	≤6%
Grain size < 100 μm	min 90%
Grain size < 63 μm	min 65%
Bulk density	about 300 g/L

^1^ Test conditions: 20 rpM, 1,9% solution, 20 °C.

**Table 3:** Physical and chemical properties of the used starch ether.

Polymer Type	Starch Ether (SE)
Appearance	Whitish or yellowish powder
Viscosity ^1^	1500–3000 mPa·s
Humidity	≤10%
Origin of the ether	Potato starch

^1^ Test conditions: 10% solution.

**Table 4:** Mortar mix proportions of all samples.

Mortar	Cement (g)	Fine Aggregate (g)	Polymer admixture (g)		Water (g)
			CE ^1^	SE ^2^	
REF	450	1350	-	-	600
CE1	450	1350	1	-	600
CE2	450	1350	2	-	600
CE3	450	1350	3	-	600
CE4	450	1350	4	-	600
SE1	450	1350	-	1	600
SE2	450	1350	-	2	600
SE3	450	1350	-	3	600
SE4	450	1350	-	4	600

^1^ Cellulose ether and ^2^ starch ether.

**Table 5:** Water retention results of all mortars.

Mortar	WRV10	WRV20	WRV30	WL
	(%)	(%)	(%)	(%)
REF	80.3	74.2	71.1	28.9 ^1^
CE1	87.7	84.9	82.4	17.6
CE2	96.1	93.8	92.5	7.5
CE3	98.9	97.3	96.8	3.2
CE4	99.5	99.4	99.2	0.8
SE1	88.1	82.9	76.8	23.2
SE2	84.6	81.3	78.2	21.8
SE3	85.8	85.1	84.5	15.5
SE4	83.8	83.5	81.5	18.5

^1^ Water loss—difference between 100% and WRV30.

**Table 6:** Heat of hydration of tested pastes.

Symbol of Paste	Time of First Maximum (h)	Heat after Hours of Hydration (J/g)					
		12	24	36	48	60	72
REF	12 h 30 min	100.10	198.64	249.69	274.17	290.90	304.06
CE1	14 h 2 min	80.06	175.99	228.96	255.12	272.50	285.89
CE2	15 h 51 min	78.70	171.17	226.77	254.65	272.70	286.46
CE3	18 h 51 min	76.67	163.95	223.50	253.92	272.98	287.32
CE4	19 h 32 min	76.49	158.09	221.53	255.00	275.15	290.03
SE1	13 h 3 min	69.11	152.92	198.67	222.24	238.59	251.75
SE2	13 h 13 min	69.21	140.16	180.33	203.02	219.69	233.59
SE3	12 h 52 min	69.31	127.39	161.98	183.80	200.79	215.43
SE4	13 h 20 min	74.71	114.84	140.22	157.12	170.42	181.66

**Table 7:** Degree of hydration of tested pastes.

Symbol of Paste	Degree of Hydration (-)					
	12 h	24 h	36 h	48 h	60 h	72 h
REF	32.92	65.33	82.12	90.17	95.67	100.00
CE1	26.33	57.88	75.30	83.90	89.62	94.02
CE2	25.88	56.30	74.58	83.75	89.68	94.21
CE3	25.21	53.92	73.50	83.51	89.78	94.49
CE4	25.16	51.99	72.86	83.87	90.49	95.39
SE1	22.73	50.29	65.34	73.09	78.47	82.80
SE2	22.76	46.10	59.31	66.77	72.25	76.82
SE3	22.79	41.90	53.27	60.45	66.03	70.85
SE4	24.57	37.77	46.12	51.67	56.05	59.74

**Table 8:** Assessment of the R^2^ coefficient and the relationship between the amount of cellulose ether and the properties of tested mortars.

Mortar Property	Equation	R^2^	Correlation	Dependence
Flow	y = −4.8x + 162.8	0.883	high	considerable
Cone Penetration	y = 0.07x + 5.08	0.078	low	clear, but small
Air Content	y = 4.3x + 4.5	0.983	high	considerable
Bulk Density	y = −80x + 1868	0.996	high	considerable
WRV10	y = 4.96x + 77.62	0.897	high	considerable
WRV20	y = 6.277x + 71.097	0.912	high	considerable
WRV30	y = 7.06x + 67.22	0.924	high	considerable

**Table 9:** Assessment of the R^2^ coefficient and the relationship between the amount of cellulose ether and the properties of tested pastes.

Paste Property	Equation	R^2^	Correlation	Dependence
Initial Setting Time	y = 57.5x + 286.5	0.951	high	considerable
Final Setting Time	y = 70.5x + 361.5	0.957	high	considerable
Setting Time	y = −14.5x + 88	0.922	high	considerable

**Table 10:** Assessment of the R^2^ coefficient and the relationship between the amount of starch ether and the properties of tested mortars.

Mortar Property	Equation	R^2^	Correlation	Dependence
Flow	y = −6.6x + 160.6	0.852	high	considerable
Cone Penetration	y = −0.57x + 4.48	0.526	high	considerable
Air Content	y = 0.8x + 5.7	0.941	high	considerable
Bulk Density	y = −10x + 1854	0.893	high	considerable
WRV10	y = 0.457x + 83.15	0.064	low	clear, but small
WRV20	y = 2.087x + 75.13	0.601	high	considerable
WRV30	y = 2.85x + 69.87	0.791	high	considerable

**Table 11:** Assessment of the R^2^ coefficient and the relationship between the amount of starch ether and the properties of tested pastes.

Paste Property	Equation	R^2^	Correlation	Dependence
Initial Setting Time	y = 365.5x − 59.5	0.997	high	considerable
Final Setting Time	y = 393x + 4	0.998	high	considerable
Setting Time	y = 27.5x + 91	0.961	high	considerable

**Table 12:** Comparison of properties of cellulose ether and starch ether on tested mortars.

Mortar Property	Cellulose Ether		Starch Ether	
	Tendency	Intensity ^1^	Tendency	Intensity ^1^
Flow	decreasing	++	decreasing	+++
Cone Penetration	decreasing	+	decreasing	+++
Air Content	growing	+++	growing	+
Bulk Density	decreasing	++	decreasing	+
WRV10	growing	+++	growing	+
WRV20	growing	+++	growing	++
WRV30	growing	+++	growing	++

^1^ + low impact; ++ high impact; +++ very high impact.

**Table 13:** Comparison of properties of cellulose ether and starch ether on tested pastes.

Mortar and Paste Property	Cellulose Ether		Starch Ether	
	Tendency	Intensity ^1^	Tendency	Intensity ^1^
Initial Setting Time	delaying	+	delaying	+++
Final Setting Time	delaying	+	delaying	+++
Setting Time	drawing out	+	drawing out	+++
Cumulative Heat	decreasing	+	decreasing	+++
Degree of hydration	decreasing	+	decreasing	+++

^1^ + low impact; +++ very high impact.

## Data Availability

No new data were created or analyzed in this study. Data sharing is not applicable to this article.
